# Listas de espera na atenção ambulatorial especializada: reflexões sobre um conceito crítico para o Sistema Único de Saúde

**DOI:** 10.1590/0102-311XPT220724

**Published:** 2025-06-20

**Authors:** Elaine Maria Giannotti, Marília Louvison, Arthur Chioro

**Affiliations:** 1 Universidade Federal de São Paulo, São Paulo, Brasil.; 2 Universidade de São Paulo, São Paulo, Brasil.

**Keywords:** Listas de Espera, Atenção Secundária à Saúde, Acesso aos Serviços de Saúde, Sistema Único de Saúde, Waiting Lists, Secondary Care, Access to Health Services, Unified Health System, Listas de Espera, Atención Secundaria de Salud, Acceso a los Servicios de Salud, Sistema Único de Salud

## Abstract

O debate sobre a oferta e organização dos serviços da atenção ambulatorial especializada (AAE) no Sistema Único de Saúde (SUS) e a necessidade de redução dos tempos de espera para o acesso a um cuidado integral e de qualidade tem se constituído em uma das mais importantes agendas para gestores do SUS e pesquisadores da área de Política, Planejamento, Gestão e Avaliação em Saúde, e envolve, também, vários segmentos da sociedade civil. Trata-se de uma temática com déficit de formulação teórica, normativa e fundamental para a consolidação do SUS como sistema integral e de acesso universal. Recentemente, alguns avanços para o enfrentamento desse grave problema foram implementados, como a instituição da Política Nacional da Atenção Especializada em Saúde, pelo Ministério da Saúde, e experiências bem-sucedidas implementadas por algumas gestões estaduais e municipais. O presente artigo, de caráter ensaístico, foi produzido a partir das reflexões teóricas e das vivências de seus autores como gestores nas diferentes esferas de gestão do SUS. Apresenta quatro apontamentos sobre os desafios relacionados à temática das listas de espera. O primeiro, busca analisar criticamente se as extensas filas de espera são mesmo o principal problema para o acesso à AAE do SUS. A seguir, discute os desafios para monitorar os tempos de espera para o acesso à AAE. O terceiro apontamento indica e analisa dispositivos regulatórios que vêm sendo utilizados para o manejo de listas de espera com foco nas necessidades dos usuários. Por fim, o quarto apontamento trata da necessidade de produzir e garantir transparência nas listas de espera para gestores, trabalhadores e usuários.

## Abstract (en)

The debate on the supply and organization of specialized outpatient care at the Brazilian Unified National Health System (SUS) and the need to reduce waiting times for access to comprehensive and quality care has become one of the most important agendas for SUS managers and researchers in Health Policy, Planning, Management, and Evaluation and various civil society segments. This theme shows a gap in its theoretical, normative, and fundamental formulation to consolidate SUS as a comprehensive system with universal access. Some recent advances to face this serious problem have been implemented, such as the Brazilian National Policy for Specialized Health Care, of the Brazilian Ministry of Health, and the successful experiences of some state and municipal administrations. This essay was based on the theoretical reflections and experiences of its authors as SUS managers. It offers four reflections on the challenges to waiting lists. the first of which seeks to critically analyze whether long waiting lines really constitute the main problem for access to the SUS specialized outpatient care. This study then discusses the challenges to monitoring waiting times for specialized outpatient care access. The third point indicates and analyzes regulatory devices that have managed waiting lists, focusing on the needs of users. Finally, the fourth reflection addresses the need to produce and ensure transparency in the waiting lists for managers, workers, and users.

## Resumen

El debate sobre la prestación y organización de servicios de atención ambulatoria especializada (AAE) en el Sistema Único de Salud brasileño (SUS) y la necesidad de reducir el tiempo de espera para el acceso a una atención integral y de calidad han sido uno de los temas más importantes para los gestores e investigadores del SUS en el área de Política, Planificación, Gestión y Evaluación de la Salud, y también involucra a varios segmentos de la sociedad civil. Se trata de un tema que carece de formulación teórica, normativa y fundamental para la consolidación del SUS como sistema integral y de acceso universal. Recientemente se han implementado algunos avances para enfrentar este grave problema, como el establecimiento de la Política Nacional de Atención Especializada en Salud por parte del Ministerio de Salud brasileño, y experiencias exitosas implementadas por algunas administraciones estatales y municipales. Este artículo ensayístico fue elaborado a partir de las reflexiones teóricas y experiencias de sus autores como gestores en los diferentes ámbitos de la gestión del SUS. Presenta cuatro notas sobre los desafíos relacionados con el tema de las listas de espera. La primera busca analizar críticamente si las extensas filas son realmente el principal problema para acceder a AAE del SUS. A continuación se analizan los desafíos en el monitoreo de los tiempos de espera para el acceso a AAE. La tercera nota indica y analiza las disposiciones reglamentarias que se han utilizado para la gestión de las listas centradas en las necesidades de los usuarios. Por último, la cuarta nota aborda la necesidad de producir y garantizar la transparencia en las listas de espera para gerentes, trabajadores y usuarios.

## Introdução

Pesquisas de opinião, de forma recorrente, demonstram que a dificuldade para garantir acesso à atenção ambulatorial especializada (AAE) - com ênfase às consultas médicas, exames, cirurgias e procedimentos especializados - constitui-se em um dos mais graves problemas do Sistema Único de Saúde (SUS) ^1^. Trata-se de um quadro crítico que acaba se constituindo na face visível do SUS, reverberado pela imprensa, indicativo do sofrimento dos usuários que não conseguem ou têm enormes dificuldades para garantir acesso aos serviços especializados e aguardam seu agendamento em listas de espera. É também um objeto frequente de questionamentos por parte de órgãos de controle, resultando em ações judiciais que, muitas vezes, determinam a priorização do atendimento de uma pessoa em detrimento de outras, aprofundando o padrão de iniquidade já existente.

Deve-se considerar que a demanda para a AAE é frequentemente estimulada muito mais por pressões decorrentes do mercado do que por reais necessidades dos usuários ^2^. Além disso, por vezes, no momento do agendamento, algumas pessoas já foram atendidas em outro local e não necessitam mais de atendimento ou não comparecem ao serviço ^3^.

É sabido, também, que a espera demasiada de pacientes com doenças crônicas ou algumas situações agudas pode levar à perda de oportunidade terapêutica e um consequente prognóstico desfavorável. O incipiente uso de alguns protocolos de classificação de risco e vulnerabilidades, associado às fragilidades na gestão das listas de espera, agravam esse problema. Ademais, o subfinanciamento histórico do SUS restringe o acesso e reduz a oferta de ações e serviços de saúde, a partir das necessidades identificadas nos territórios ^4^.

O tempo de espera elevado para o acesso à AAE tem se constituído como um importante desafio para os sistemas de saúde universais, considerando o desbalanço existente entre oferta e demanda. Esses tempos de espera foram agravados em todo o mundo com o represamento de consultas, exames e cirurgias eletivas suspensas em função da pandemia de COVID-19 ^5^. Vários países com excessivos tempos de espera para serviços especializados têm apresentado experiências de priorização de filas para transplantes, cirurgias, exames e terapias de maior complexidade ^6^. A necessidade de priorizar os casos mais graves, com a intervenção em tempo oportuno, traz benefícios às pessoas e ao sistema, produzindo justiça e equidade, levando os tomadores de decisão a organizarem as chamadas listas de espera e monitorar o tempo de espera ^7^. No entanto, a carência de medidas de promoção, prevenção e diagnósticos precoces amplia o número de futuros casos graves, constituindo-se um perverso e indesejável círculo vicioso.

A lista de espera da AAE não pode ser um lugar de não cuidado onde os usuários são empilhados até um futuro incerto, marcado pela incerteza sobre em que momento serão atendidos, sem qualquer processo de acompanhamento por parte da unidade solicitante, da unidade executante e das centrais de regulação. Entre as limitações existentes nos sistemas de saúde, destacam-se as fragilidades na gestão das listas de espera e a insuficiência de formação profissional para essa tarefa, que pressupõe compreensão de determinados conceitos e conhecimentos operacionais ^8^. A utilização de dispositivos regulatórios para esse acompanhamento, que colaborem na produção do cuidado integral e na redução dos tempos de espera, sobretudo para os casos mais graves, precisa ser fortalecida, assim como iniciativas que propiciem a criação de espaços de diálogo entre a atenção primária em saúde (APS) e a AAE, o uso de ferramentas de saúde digital e estratégias de matriciamento para a construção de arranjos de compartilhamento do cuidado. 

O cuidado integral em saúde, entendido como tratar, respeitar, acolher e atender o ser humano em seu sofrimento - não somente do ponto de vista clínico, mas também considerando sua vulnerabilidade social ^9^ - constitui-se em um dos principais objetivos do SUS. Apesar de algumas iniciativas federais, como as políticas adotadas para organização da assistência de alta complexidade no SUS para terapia renal substitutiva, oncologia, doenças cardiovasculares, dentre outras, levadas a cabo entre 2003 e 2005 - no primeiro governo Luiz Inácio Lula da Silva -, e a implantação das redes temáticas (Cegonha, Atenção Psicossocial, Cuidados da Pessoa com Deficiência, Atenção às Urgências e Atenção à Saúde das Pessoas com Doenças Crônicas) - no governo Dilma Rousseff -, a ampliação do acesso à atenção especializada em saúde e a qualificação do cuidado permanecem como grandes desafios na implantação do SUS.

Nesse sentido, recentemente o Ministério da Saúde adotou a atenção especializada como uma agenda prioritária e lançou a Política Nacional de Atenção Especializada em Saúde (PNAES) ^10^. Entre as diretrizes da PNAES pode-se destacar a promoção de um modelo de atenção centrado nas necessidades de saúde das pessoas e no cuidado ao usuário, a garantia de coordenação do cuidado e continuidade assistencial e o fortalecimento de um novo modelo de regulação, centrada do usuário e produtor de cuidados, que promova a corresponsabilização e comunicação entre equipes demandantes, ofertantes e usuários. A partir dessa política, o Ministério da Saúde vem desenvolvendo um conjunto de ações com alocação de volumosos recursos para o Programa Nacional de Redução das Filas de Espera de Procedimentos Cirúrgicos Eletivos ^11^ e o Programa Mais Acesso a Especialistas ^12^. Esse último visa ampliar e tornar mais rápido o acesso dos pacientes a consultas ambulatoriais e exames especializados, a partir de uma fila única e uma definição de tempo máximo para o diagnóstico, utilizando dispositivos para coordenação e navegação do cuidado.

O presente estudo, de caráter ensaístico, se propõe a aportar reflexões que possam contribuir com o debate contemporâneo da AAE no SUS, com vistas à garantia do cuidado e do acesso equânime e oportuno aos serviços de saúde. Para tal, toma como objeto de análise as listas de espera para AAE, considerando que o problema a ser enfrentado não é o tamanho das listas em si, mas os elevados tempos que as pessoas permanecem aguardando atendimento e os incipientes mecanismos de gestão e falta de transparência dessas listas de espera para promoção do cuidado compartilhado.

Para tanto, foram produzidas contribuições teóricas sobre aspectos relacionados às listas de espera da AAE no SUS, concebidas a partir da revisão da incipiente literatura existente sobre o tema ^13^ e das vivências dos autores como pesquisadores e gestores que há três décadas se dedicam ao tema, com experiências nas esferas federal, estadual e municipal do SUS. 

Para o desenvolvimento das análises aqui contidas, propomos quatro apontamentos centralmente relacionados à temática das listas de espera da AAE: (i) As extensas filas de espera para o acesso à AAE são mesmo o principal problema do SUS?; (ii) O tempo de espera para o acesso à AAE em análise; (iii) Dispositivos regulatórios para gestão de listas de espera: conhecer para cuidar; e (iv) Algumas reflexões sobre transparência das listas de espera da AAE.

## As extensas filas de espera para o acesso à AAE são mesmo o principal problema do SUS?

Filas são uma maneira democrática de garantir acesso a um serviço ou produto, a partir de critérios como ordem de chegada, grupos populacionais, classificação de risco, dentre outros. As filas podem ser classificadas como presenciais e não presenciais, ou como únicas ou múltiplas. Têm um caráter dual: por um lado, o cidadão tem que se submeter à espera, quando o desejo é ter atendimento imediato; por outro, saber que está numa fila e que será atendido em algum momento pode lhe conferir algum grau de segurança no processo ^14^.

A teoria de filas é um método estatístico que estuda a formação de filas e estima os tempos de espera a partir de suas propriedades, com o uso de fórmulas matemáticas, identificando padrões de comportamento do funcionamento da fila, no sentido de apoiar o dimensionamento dos serviços e evitar desperdícios e gargalos ^15^. É utilizada para estimar as demoras para o acesso a um serviço prestado a clientes que chegam ao acaso a um local onde o serviço não pode ser oferecido prontamente. A análise de um sistema de filas estuda elementos como o regime de chegada, regime de serviço e disciplina da fila, fornecendo modelos que ajudam a prever o desempenho dos sistemas de serviços, analisando o tempo médio de espera e a utilização dos recursos, garantindo a eficiência operacional e a satisfação dos usuários ^16^. De acordo com Fogliatti & Matos ^17^, as regras de atendimento mais utilizadas são: FIFO (*first in*, *first out*) por ordem de chegada (primeiro a chegar, primeiro a sair); LIFO (*last in*, *first out*), o último a chegar é o primeiro a ser atendido; PRI (*priority service*), por ordem de prioridade; SIRO (*service in random order*), por ordem aleatória.

Para alguns autores, o cidadão em fila perde sua individualidade, tornando-se apenas um número ^14^, o que deve ser fortemente evitado, sobretudo quando nos referimos a filas para o acesso a um cuidado em saúde. Na saúde, as filas devem ser geridas por listas de espera, envolvendo um sistema gerencial transparente e organizado por riscos, onde o PRI deveria ser o mecanismo de atendimento a ser utilizado ^18^. As filas presenciais na saúde ocorrem normalmente nas portas de urgências dos serviços, que tem se utilizado de métodos de classificação de risco para priorização dos atendimentos. O Ministério da Saúde, por meio do Programa de Apoio ao Desenvolvimento Institucional do SUS (Proadi), executado em parceria com o Hospital Sírio-Libanês, tem induzido o uso do Sistema Lean nas emergências do SUS. A metodologia Lean é uma filosofia de melhoria de processos baseada em tempo e valor, desenhada para assegurar fluxos contínuos e eliminar desperdícios e atividades de baixo valor agregado, com o objetivo de diminuir o tempo de espera no atendimento nas portas de emergência, redução do tempo de permanência e aumentar o giro dos leitos ^19^. 

Nas unidades básicas de saúde (UBS), a demanda espontânea é uma parcela importante dos atendimentos e, a depender da maneira como se organizam, também podem gerar filas presenciais de pessoas aguardando uma senha para agendar uma consulta. Em todos os serviços de saúde de porta aberta, estratégias de gestão da demanda, no sentido de adequar a oferta para garantir o atendimento ao menor tempo possível, são um grande desafio. A teoria de filas, que considera as variáveis que se apresentam de modo aleatório, indica que a melhor forma de agendamento na APS é por blocos de hora ^1^, para otimizar os recursos e evitar longas esperas. O agendamento por bloco de hora faz com que as pessoas agendadas não precisem esperar muito tempo para seu atendimento, ao mesmo tempo em que possibilita o encaixe da demanda espontânea que necessita de um atendimento imediato. Cirino et al. ^20^, em estudo de uma UBS do Município de Diadema (São Paulo), apontam que a estratégia de acesso avançado permite que os serviços se organizem de forma que os usuários sejam atendidos no mesmo dia ou em até 48 horas, conforme a demanda se apresente como urgência, rotina ou prevenção. Esse método se mostrou eficaz na redução do absenteísmo e das filas, favorecendo a continuidade do cuidado. O acolhimento com escuta qualificada também tem se mostrado uma estratégia eficaz na identificação de prioridades, evitando longas esperas nas situações de maior risco e vulnerabilidade ^21^. 

Na AAE, as filas normalmente são não presenciais, conformando listas de espera que não são formadas ao acaso, pois são geradas pelo próprio sistema de saúde a partir da análise da demanda e da adoção de critérios que definem esses encaminhamentos. Respaldados pela Lei de Little da Teoria das Filas, Pazin-Filho et al. ^22^ afirmam que se deve distinguir os termos lista e fila, onde a lista é um dos componentes da fila, que poderá ser mensurado multiplicando-se o valor da velocidade de entrada de pacientes na lista pelo tempo médio de espera para o atendimento. No entanto, cabe ressaltar que a questão das filas na saúde, sobretudo na AAE, vai além de uma organização espacial ou matemática, estendendo-se às Ciências Humanas e Sociais ^14^. 

Para acessar serviços da AAE os usuários são encaminhados por uma unidade solicitante, mediados ou não por uma central de regulação, para um serviço especializado que irá organizar a oferta disponível monitorando consultas novas, necessidade de exames complementares, retornos e altas. Quando não há possibilidade de agendamento imediato a partir da solicitação, como geralmente ocorre em um sistema cuja demanda tende a ser maior do que a oferta, os usuários são inseridos em listas de espera, aguardando por um ou mais procedimentos. Portanto, não se trata de filas, mas de listas de espera, em que a gestão e os serviços devem se debruçar para compreender as singularidades e necessidades de cada situação para a garantia de um cuidado integral e acesso em tempo oportuno, evitando o “empilhamento” dos usuários. 

Nos sistemas de saúde é aceitável uma espera para o acesso a procedimentos eletivos, pois o contrário pode significar que o sistema está sempre de prontidão para atender qualquer demanda a qualquer momento, o que não é viável do ponto de vista econômico ^3^, visto que os procedimentos eletivos são aqueles onde é possível aguardar, sem comprometer ou colocar em risco a saúde dos usuários. Vários países com sistemas de saúde que garantem acesso universal à saúde adotam listas de espera como mecanismo de regulação de acesso à atenção especializada. O problema em si não é a existência dessas listas, mas quando elas se tornam um amontoado de registros, que sistemas de informações e os próprios serviços desconhecem as necessidades dos usuários e suas singularidades.

No SUS, as listas de espera da AAE estão dispersas em vários sistemas que não interoperam entre si, há duplicidades e mantém como demanda não atendida pessoas que não mais necessitam dos serviços solicitados. Muitas vezes o critério cronológico é o único utilizado para o agendamento. Há que se compreender, todavia, que o tamanho da lista não é em si um problema. Listas com muitos nomes aguardando agendamento podem refletir a grande oferta para determinados tipos de serviços. As listas são dinâmicas, já que diariamente entram novos nomes e saem outros tantos. Por exemplo, uma lista de espera com 10 mil pessoas aguardando por um exame é um problema a ser enfrentado? Se a oferta for de mil exames ao mês, podemos inferir que sim, pois as pessoas terão que aguardar em média 10 meses para o atendimento, ao passo que se a oferta for de 10 mil exames ao mês, a espera provavelmente será pequena e não se apresentará como problema. Em contrapartida, uma lista com apenas 10 pessoas aguardando um procedimento em que não há oferta no território pode ser muito mais problemática do que uma lista com 10 mil ou 100 mil pessoas.

Monitorar se as listas estão aumentando ou diminuindo não permite fornecer subsídios para o enfrentamento das dificuldades de acesso à AAE. Longas filas cirúrgicas, por exemplo, podem servir de argumento para conseguir mais consultas para cada especialidade, muitas vezes refratárias a um gerenciamento centralizado das listas, que tem a possibilidade de redução dos tempos de espera, com a retirada de pacientes já operados ou sem indicação e realização da grade cirúrgica para evitar perdas de agendamentos em casos de cancelamento ^22^. Ademais, a lista pode diminuir porque a oferta tornou-se tão escassa que os profissionais simplesmente deixam de realizar encaminhamentos. Ou, ao contrário, a lista pode ampliar significativamente de tamanho quando um novo serviço é implantado no território, ampliando sua oferta. Dessa forma, o objetivo não deve ser diminuir ou zerar as filas da AAE, mas estar centrado na redução dos tempos de espera. Advogamos, assim, que a análise de uma lista de espera deva considerar como indicador de monitoramento, fundamentalmente, os tempos de espera e não o tamanho da lista.

## O tempo de espera para o acesso à AAE em análise

Com o envelhecimento da população e o aumento da prevalência de doenças crônicas, a problemática do tempo de espera já se coloca como desafiadora para metade dos países da OCDE (Organização para a Cooperação e Desenvolvimento Econômico) ^3^ ^,^ ^7^ ^,^ ^23^ ^,^ ^24^. O sistema de saúde espanhol definiu tempos máximos de espera nas listas com publicidade dessas informações, embora seja sabido que muitas agendas ainda estão sob o controle dos hospitais ^25^. Nas Comunidades Autônomas desse país, estudadas por Conill et al. ^7^, foram identificados tempos máximos de espera para cirurgias eletivas, consultas e exames especializados com o estabelecimento de circuitos preferenciais para determinados agravos. Outros países, como Inglaterra, Suécia, Nova Zelândia, Noruega, Países Baixos, Itália e Dinamarca também adotam mecanismos para garantia de tempos máximos de espera, a maioria condicionado a critérios de gravidade da doença ou definindo uma proporção de pacientes que deveriam ser atendidos em um determinado período de tempo ^3^.

O SUS convive com um subfinanciamento crônico e oferta subdimensionada ^4^, o que acentua o problema dos tempos de espera para AAE. O quadro é agravado pelo acúmulo frequente de registros nas listas de espera de casos sem hipótese diagnóstica ou classificação de risco, com nomes duplicados, ou pela manutenção de usuários que por vários motivos já não necessitam mais daquele recurso. Como os sistemas de informação não são integrados ^26^, é comum situações em que os usuários passam de uma fila para outra. São inseridos em uma fila de consulta para avaliação cirúrgica ou para iniciar tratamento oncológico, mediado pelas centrais de regulação, que deveriam monitorar essas listas, mas que depois “perdem” esse paciente, que passa a compor listas internas de cada serviço para realizar o procedimento cirúrgico necessário ou iniciar o tratamento indicado. As listas para consultas e exames, em geral, podem e devem ser monitoradas por sistemas internos que regulem as ofertas sob gestão de cada esfera de governo. Todavia, como os sistemas não interoperam, os encaminhamentos realizados para serviços estaduais ou por outros municípios dificilmente recebem o devido acompanhamento pelos solicitantes.

A Organização Mundial da Saúde (OMS) aponta que os longos tempos de espera para consultas e exames especializados são uma medida para identificar um sistema de saúde como responsivo ^23^. No SUS, os prolongados tempos de espera para esses procedimentos eletivos estão entre os principais problemas para o alcance da integralidade no cuidado. As normativas legais sobre tempos de espera, no Brasil, se referem apenas aos pacientes oncológicos, para os quais há prazo determinado de 60 dias para o início do tratamento a partir do diagnóstico ^27^ e de 30 dias para os exames diagnósticos a partir da solicitação médica ^28^. No entanto, os sistemas de informação do SUS têm dificuldades para identificar esses prazos, dificultando o monitoramento da aplicação dessas normas legais. Estudo realizado para analisar os fatores associados ao tempo para início do tratamento do câncer de cólon e reto no Brasil ^29^, apontou que 22% e 35% dos pacientes tiveram respectivamente atraso para início do tratamento acima dos 60 dias, incluídos no estudo pacientes tratados em serviços particulares. Concluiu-se que não houve redução desses tempos após a *Lei nº 12.732* ^27^, havendo uma distribuição desigual de serviços nas regiões e estados brasileiros e falta de gestão causando problemas nos encaminhamentos e dificuldades nos agendamentos de exames diagnósticos. Estudo ^29^ aponta diferenças importantes nos tempos de espera para início do tratamento de câncer de mama, colo de útero e próstata relacionados à idade, raça/cor da pele, estado conjugal, escolaridade, região de residência, tipo de tratamento necessário, estadiamento e distância entre a residência e o hospital.

Em abril de 2024, com a instituição do Programa Mais Acesso a Especialistas ^12^, o Ministério da Saúde definiu um prazo de 30 a 60 dias para conclusão da etapa referente ao diagnóstico de algumas Linhas de Cuidado prioritárias, a ser cumprido pelos estabelecimentos de saúde que se habilitarem no programa.

Para o monitoramento dos tempos de espera faz-se necessário estabelecer métricas ou indicadores para mensurá-los. A média dos tempos de espera para cada procedimento pode ser um bom indicador, que pode ser acrescido do percentual de pessoas em relação ao total, em espera por determinado procedimento, por um tempo definido como aceitável. No entanto, é importante ficar atento aos valores dos desvios padrão dessas médias, pois quanto maior o desvio padrão, maior será a diferença entre os tempos máximos e mínimos, o que requer uma análise detalhada de que tipo de situação está influenciando o valor da média para patamares muito elevados. Essas situações requerem uma análise individualizada dos casos com tempos muito elevados e suas motivações para pensar em alternativas visando o adequado manejo desses casos. O uso da mediana também pode ser indicado, por não ser afetada por valores extremos.

Há uma dificuldade para mensurar os tempos de espera, que reflete a baixa capacidade de gestão para o enfrentamento desse problema. Os tempos podem ser divididos conforme as diferentes situações que o usuário tem que enfrentar, como o intervalo de tempo entre o encaminhamento do solicitante e o agendamento, entre o agendamento e execução do procedimento, ou ainda o tempo total entre o encaminhamento e a execução da consulta ou exame ^21^. Os tempos também podem ser medidos, conforme proposto pelo Programa Mais Acesso a Especialistas, pelo tempo entre o encaminhamento e a finalização do diagnóstico, independentemente de quantos exames sejam necessários. Esse olhar tende a produzir menos fragmentação do cuidado, pois busca definir um tempo mínimo para um determinado trecho da condução do usuário pela linha de cuidado e não para cada procedimento isoladamente. Ainda mais porque a lógica até então reinante, herdada desde os áureos tempos de INAMPS (Instituto Nacional de Assistência Médica da Previdência Social), privilegia a remuneração dos prestadores de serviços por procedimentos isoladamente ^30^. Na proposição do Programa Mais Acesso a Especialistas, as listas estão inicialmente focadas em pacotes de procedimentos diagnósticos, chamados de Oferta de Cuidados Integrados (OCI), definidos para cada linha de cuidado. Evidentemente, pressupõe grandes mudanças no processo regulatório e no modelo de remuneração, com o objetivo de obter registro agregado e visões unificadas das listas municipais, regionais e estaduais, que contém informações essenciais e singulares sobre as demandas para a AAE nas diferentes regiões de saúde do país.

Pensar em formas de monitoramento dos tempos de espera é um grande desafio, tendo em vista que os tempos médios podem mascarar grandes problemas e desigualdades no acesso. Ademais, olhar para cada procedimento de forma isolada não traduz a necessidade de um cuidado integral, em rede, e em tempo oportuno para quem mais necessita.

## Dispositivos regulatórios para gestão de listas de espera: conhecer para cuidar 

As listas de espera da AAE no SUS podem tanto garantir acesso oportuno como restringi-lo, a depender da maneira como é feita sua gestão. Atualmente, essas listas são múltiplas para o mesmo tipo de procedimento, sem padronização de nomenclaturas. É comum encontrarmos encaminhamentos indevidos ou repetidos ^31^ e mais de uma lista para o mesmo procedimento ^32^. Solicitantes e executantes deveriam se ocupar em conhecer as pessoas que estão na lista e pensar em estratégias conjuntas para seu cuidado, mas isso raramente ocorre. A UBS deveria acompanhar os pacientes que foram por ela inseridos em listas de espera para especialidades e identificar os casos que poderiam ser atendidos e resolvidos na própria UBS, individualmente ou mediante discussão de caso com a equipe ou matriciamento com outros profissionais da rede ^33^. Nesse sentido, a atuação dos profissionais da APS na qualificação dos encaminhamentos é fundamental para o alcance da integralidade do cuidado ^23^.

A implantação de estratégias para redução de encaminhamentos indevidos, aliada a processos de matriciamento com a AAE e educação permanente, têm se mostrado eficaz para qualificação dessas listas, com vistas à redução dos tempos de espera. Estudo realizado por Brainard et al. ^34^ demonstrou o sucesso de uma experiência realizada no leste da Inglaterra, onde foi implantado um programa em que a atenção primária recebeu um dermatoscópio e um aplicativo de celular para envio de imagens de pacientes com problemas dermatológicos, para análise e diálogo com dermatologistas consultores, de maneira a reduzir o encaminhamento para atenção secundária. Esse estudo demonstrou que 95% dos diálogos foram resolvidos em 36 horas, com alto grau de satisfação dos médicos e pacientes.

No Rio Grande do Sul, a relação entre a teleassistência e os médicos da APS permitiu reduzir em 50% a fila por procedimentos de endocrinologia ^1^. O mesmo ocorreu na reumatologia em Uberlândia (Minas Gerais), a partir da elaboração de protocolo clínico de manejo de doenças reumáticas, com capacitação das equipes de APS na estratificação de riscos e no manejo dessas doenças. O resultado indica redução de 78% de pacientes em lista de espera, sem a necessidade de contratação de mais especialistas ^1^.

A redução das taxas de perdas primárias e absenteísmo com vistas à otimização da oferta existente também é ação importante. O planejamento da oferta a partir das reais necessidades do território pode reduzir perdas primárias ocasionadas pela falta de demanda para aquele serviço. Também se torna necessário conhecer os motivos que levam o usuário a não comparecer à consulta ou exame (absenteísmo) e pensar em ações para sua redução, como, por exemplo, lembrá-lo do agendamento com alguns dias de antecedência, não proceder o agendamento em data ou horários que sabidamente o usuário não poderá comparecer, fazer uma orientação detalhada sobre o preparo do exame e sua importância e prover o transporte sanitário para o usuário que necessita. No entanto, o absenteísmo, diferentemente da perda primária, sempre ocorrerá em algum grau, à medida em que não depende exclusivamente da organização dos serviços e do sistema de saúde. As razões podem estar relacionadas a questões de ordem pessoal. Nesse sentido, estratégias como *overbooking* têm sido utilizadas para ampliar o uso dos recursos públicos, considerando as dificuldades de controlar todos os fatores que levam ao não comparecimento dos usuários ^35^.

A gestão de listas de espera também passa pelo aprimoramento de protocolos de regulação que orientem os encaminhamentos da APS e por critérios de classificação de risco definidos com base em evidências científicas, para priorização dos usuários que mais necessitam. Barbosa ^3^ aponta a escassez de estudos sobre filas de espera e acrescenta que as propostas passam majoritariamente pelo aumento da oferta ou implantação de mecanismos de restrição da demanda. A utilização de protocolos para definição de prioridades tem sido a estratégia de vários países, a partir de dimensões como: gravidade da doença e a possibilidade de sua progressão, intensidade da dor e limitação para execução de atividades de vida diária com autonomia ^3^. No SUS, entretanto, a adoção dessa prática ainda parece ser bastante tímida, pois as equipes ainda pouco classificam risco ou monitoram suas listas de espera ^32^. 

De acordo com a Comissão Intergestores Bipartite do Estado de São Paulo ^36^, aproximadamente 4,6 milhões de usuários estavam em listas de espera para consultas especializadas, exames diagnósticos e procedimentos cirúrgicos eletivos no Cadastro de Demanda por Recurso da ferramenta Sistema Informatizado de Regulação do Estado de São Paulo (SIRESP), utilizada pela Central de Regulação de Ofertas de Serviços de Saúde (CROSS), sendo que 310 mil nomes foram inseridos entre 2007 a 2019, 195 mil em 2020, 285 mil em 2021, 710 mil em 2022, 2 milhões em 2023 e 1,1 milhão em 2024. Dos recursos solicitados, 43% eram destinados a consultas especializadas, 48% para exames diagnósticos e 9% para avaliação cirúrgica e procedimentos cirúrgicos eletivos. Além de um tempo de espera excessivo, esses números deixam claro que não há um processo de gestão dessas listas, nem pelo estado nem pelos municípios, que inseriram os nomes dos usuários na listagem sem monitoramento posterior.

Para promover um cuidado em saúde é imperioso conhecer quem são os usuários que estão na fila, qual seu agravo, como chegaram a essa fila, qual o tempo de espera, quais cuidados podem ser oferecidos enquanto aguardam na fila e qual o grau de prioridade para realizar seu agendamento. Isso é o que aqui denominamos de fazer a gestão da lista. Para tanto, é necessário investimento e organização em todo o sistema de saúde de modo a envolver a gestão e todos os serviços que compõem a rede.

Para Mendes ^1^, os problemas das filas da AAE manifestam-se nos campos da oferta, da demanda e da logística e há uma tendência no SUS de aumento de oferta sem a concomitante racionalização dessa oferta, da demanda e da logística. Sugere adoção de critérios de inclusão e exclusão das pessoas nas filas, de comunicação na APS e na atenção especializada e ações de nacionalização no campo da demanda para o enfrentamento das filas no SUS. A regulação pode auxiliar na gestão dessas listas, bem como na escuta das singularidades e na definição de caminhos e fluxos entre os serviços da rede. Bertussi et al. ^37^ demonstraram a potência do uso de dispositivos, como consultas compartilhadas entre APS e AAE, discussões de casos e matriciamento, utilizados em diferentes situações. 

O monitoramento das listas de espera pode fornecer importantes subsídios para ações de planejamento, como mudança no modelo de atenção, adoção de novos dispositivos para o cuidado e ajustes em contratos. A qualificação dessas listas passa por um diagnóstico da oferta da rede e das necessidades da população, identificação de encaminhamentos desnecessários para AAE, capacitação e orientação para a condução dos casos na APS, criação de espaços de discussão entre as equipes, aliados a outras estratégias para promover a articulação entre APS e AAE, com possibilidade de telematriciamento entre o médico da APS e o médico especialista para a condução do caso e, a médio prazo, passa por uma discussão acerca da formação e de mudanças significativas na prática médica. 

A identificação de casos que aguardam em listas de espera por um serviço especializado também é estratégia importante para o cuidado e o acesso oportuno. Por mais que um intervalo de tempo seja aceitável, os usuários devem ser reavaliados pela APS com vistas à elaboração de um projeto terapêutico singular e verificação de outras possibilidades de cuidado, como atenção fisioterapêutica ou a oferta de práticas integrativas e complementares em saúde (PICS), particularmente para os casos de dor crônica e de sofrimentos psicossociais. 

Nesse sentido, o PMAE traz alguns elementos dos quais a regulação tem um papel fundamental de condução de um processo, com a potência de um cuidado mais integrador que rompa com a lógica fragmentadora expressa por uma somatória de atos ou procedimentos. Ou seja, é imperioso o investimento num projeto terapêutico que aposte não apenas na dimensão das tecnologias duras e leve-duras, centrada nos saberes profissionais, mas também na dimensão cuidadora e relacional das práticas de saúde, a partir do uso de tecnologias leves, mediante um trabalho vivo em ato ^38^.

Bertussi et al. ^37^ falam de uma “regulação a quente”, que pede encontros entre os trabalhadores de uma mesma equipe e entre equipes de diferentes serviços. Esses encontros envolvem corresponsabilização e permitem um olhar para a singularidade de cada usuário na produção do acesso e continuidade do cuidado. Ressalta-se, aqui, a importância dos mecanismos para gestão das listas e de ações a serem desenvolvidas coletivamente, a partir da criação de espaços de diálogo com as equipes de saúde e, principalmente, da escuta das necessidades singulares captadas a partir do encontro dos profissionais com os usuários.

## Algumas reflexões sobre transparência das listas de espera da AAE

Para Cavalcanti et al. ^25^, os sistemas informatizados vêm desempenhando um papel importante para o conhecimento e monitoramento das listas de espera da AAE, da definição de prioridades clínicas e do controle do absenteísmo, além de permitir maior imparcialidade e transparência na organização das agendas. Dar transparências às listas de espera, oferecendo aos usuários informações sobre a situação do pedido e o tempo de espera aproximado para o procedimento solicitado, confere segurança ao sistema de saúde, além de colaborar para a redução do clientelismo. Alguns projetos de lei em tramitação da Câmara dos Deputados e Senado Federal foram propostos para tornar obrigatória a divulgação online das listas de espera para a realização de procedimentos no SUS. No Estado de São Paulo foi aprovada lei que obriga estado e municípios a darem publicidade à ordem de espera de pacientes que aguardam a realização de procedimentos ofertados pela CROSS e unidades do SUS no âmbito do Estado ^39^. Destaque-se que essa lei, ainda sem regulamentação, não vem sendo cumprida. 

A regulação do acesso no SUS padece da falta de padronização das nomenclaturas dos procedimentos da AAE, sejam consultas, exames ou terapias. Em muitas situações, são os estabelecimentos de saúde que organizam suas agendas e conferem um nome ao procedimento que ofertam. Isso faz com que várias nomenclaturas possam ser atribuídas a um mesmo procedimento, o que impede a organização de listas municipais, regionais ou estaduais de forma fidedigna. 

Há uma dificuldade inerente à lógica regulatória para informar precisamente o lugar do paciente na fila. Primeiro porque não se trata de uma fila por ordem de chegada, mas de listas de espera, para as quais são utilizados critérios de prioridade por risco e vulnerabilidade para agendar um usuário. Assim, um cidadão que acabou de ingressar na lista pode passar à frente de outro que está aguardando há mais tempo. Dar transparência a esses critérios e protocolos de acesso é necessário para evitar privilégios. Além disso, outra questão que dificulta a informação do lugar na fila é o fato de encontrarmos diversas listas. O mesmo usuário pode se encontrar aguardando o mesmo procedimento em listas estaduais, regionais, municipais e internas aos serviços. Outro dificultador é que essas listas estão dispostas em diversos sistemas informatizados, que não interoperam entre si.

O avanço da tecnologia da informação e das redes sociais nos últimos anos, possibilitou que alguns hospitais disponibilizassem as listas em seus *sites*. No entanto, há evidências de dificuldade de interpretação dessas informações pelos usuários e órgãos de controle, em virtude das terminologias utilizadas, das formas de gerenciamento características de cada instituição e das formas utilizadas para se adequarem à Lei Geral de Proteção de Dados ^22^.

No entanto, a transparências dos dados é extremamente desejável e fornece aos usuários e equipes de saúde a informação de fácil compreensão sobre o tempo de espera aproximado poderia ser um bom começo, tanto para ampliar a transparência como para orientar o manejo adequado de cada caso durante o tempo de espera. A divulgação dessas listas de espera pode colaborar para um acesso equânime e permitir o conhecimento do usuário sobre o sistema de saúde, além de combater fraudes e privilégios ^40^. Entretanto, esse é um campo de disputas e pressões políticas, econômicas e corporativas, que podem alterar a forma de divulgação e da contagem dos tempos de espera ^7^.

Há experiências pontuais em alguns estados e municípios de divulgação eletrônica de listas de espera, muitas delas decorrentes de acordos com o Ministério Público ou de ações judiciais, o que demonstra dificuldades e resistências de gestores e profissionais de saúde para essa prática. Em revisão de literatura sobre transparência de listas de espera ^40^ ficou evidenciada que essa é uma prática incipiente no Brasil e que a transparência na administração pública se aplica mais aos dados de execução orçamentária e financeira, diferentemente de experiências de alguns países da Europa e América do Norte, que publicam na internet suas listas de espera ou definem e monitoram tempos máximos de espera dos hospitais.

Para o usuário é fundamental a transparência das listas para que consiga ter a informação sobre se, de fato, seu nome está registrado em algum sistema, qual o tempo médio para acessar o serviço desejado e a qual serviço deve se referenciar em caso de alteração do quadro clínico. Ou seja, para ele é essencial se sentir cuidado, mesmo que o acesso a alguns procedimentos possa demorar mais tempo que o desejado. Dar transparência a essas listas é um desafio que se coloca à medida que não tem sido uma prática hegemônica no SUS e porque tem o potencial de ampliar a satisfação dos usuários ^40^.

A gestão das listas de espera realizada por uma equipe multiprofissional tem potencial de oferecer transparência no processo de cuidado ao paciente eletivo, garantindo justiça e equidade nesse processo ^8^. Construir coletivamente as soluções e depois avaliar conjuntamente os caminhos percorridos, oportunizando a produção de encontros e inovando nos processos de trabalho ^37^ parece ser um caminho para fazer a gestão dessas listas com transparência, a partir das necessidades dos usuários, com vistas ao cuidado integral, em tempo oportuno.

## Conclusão

Propiciar um cuidado integral com redução dos tempos de espera para acesso à AAE é, sem dúvida, uma das questões mais importantes para a consolidação do SUS como sistema integral e de acesso universal. Quando o assunto é saúde a demanda sempre é maior que a oferta. Não se sustenta a visão simplista de que basta aumentar a oferta para construir um equilíbrio na utilização dos serviços de saúde. É necessário considerar, também, que muitas vezes a demanda é superestimada pela intensidade de pedidos de exames decorrentes da pressão de oferta produzida pelo mercado.

É importante o entendimento de que as filas para especialidades se constituem em uma listagem de pessoas que aguardam um cuidado. Portanto, o objetivo não deve ser necessariamente acabar com as filas, mas conhecê-las e geri-las, propiciando um cuidado integral, em tempo oportuno, de acordo com as necessidades dos usuários e possibilidades dos serviços. 

Compreender como as listas são formadas e adotar medidas para que nelas se mantenham apenas aqueles usuários que de fato necessitam é um primeiro passo importante para sua qualificação. Como essas listas são dinâmicas, é importante um monitoramento do ponto de vista administrativo. O solicitante deve manter contato periódico com os usuários para ter informações atualizadas sobre a necessidade do serviço que está aguardando. Esse acompanhamento é fundamental para que essa lista seja “viva” e não um mero empilhamento de usuários, representado pelo registro acumulativo de nomes. O monitoramento do ponto de vista clínico também se faz necessário, por meio do agendamento de consultas periódicas com o médico ou enfermeiro da UBS para acompanhamento, reclassificação do risco e, se for o caso, proporcionar o cuidado possível para aquele serviço. Para o agendamento dessas consultas é importante a flexibilidade das agendas das UBS. Inserir o usuário em uma lista para aguardar um procedimento não pode desobrigar a equipe da UBS do cuidado ao usuário. Pensar em alternativas terapêuticas possíveis enquanto a pessoa aguarda na lista de espera pode aliviar o sofrimento e transmitir a segurança de que ele não foi esquecido. Solicitantes e executantes se ocupando das pessoas que estão na lista de espera e adotando estratégias conjuntas para seu cuidado, com uso de teleconsultorias com os especialistas ou matriciamento, são estratégias fundamentais para qualificação do cuidado e tem potencial de reduzir o tempo de espera, sobretudo para quem mais precisa do atendimento. Essas estratégias adquiriram ainda mais importância no contexto pós pandemia de COVID-19, quando se observou um represamento das ofertas de consultas e exames diagnósticos. 

Outras medidas importantes para a gestão das listas de espera são os critérios para o agendamento, ou seja, para definir o momento de saída da lista e de acesso ao serviço esperado. Para tanto, a regulação tem se utilizado de protocolos de acesso, que indicam a gravidade ou grau de risco de forma a superar a ordem cronológica como único critério de acesso. Além disso, o desafio de dar transparência a essas listas para que o cidadão possa ter a certeza de que não foi esquecido pelo sistema e qual o tempo médio de espera, é fundamental para dar maior credibilidade ao sistema e evitar clientelismos. 

Para o enfrentamento dos problemas referentes aos excessivos tempos de espera para acesso à AAE e garantia do cuidado integral, ações de gestão das litas de espera com transparência devem ser desenvolvidas coletivamente a partir da criação de espaços de diálogo com as equipes da APS e da AAE e, principalmente, da escuta das necessidades singulares captadas a partir do encontro dos profissionais com os usuários, com o cuidado compartilhado. Um caminho diferente que pode e precisa ser desenvolvido, visando ao fortalecimento do SUS e à produção de mais saúde.
